# Controlling the response to DNA damage by the APC/C-Cdh1

**DOI:** 10.1007/s00018-015-2096-7

**Published:** 2015-12-09

**Authors:** H. Rudolf de Boer, S. Guerrero Llobet, Marcel A. T. M. van Vugt

**Affiliations:** Department of Medical Oncology, Cancer Research Center Groningen, University Medical Center Groningen, University of Groningen, Groningen, The Netherlands

**Keywords:** Cell cycle, DNA damage, Checkpoint, E3 ligase, Cyclosome

## Abstract

Proper cell cycle progression is safeguarded by the oscillating activities of cyclin/cyclin-dependent kinase complexes. An important player in the regulation of mitotic cyclins is the anaphase-promoting complex/cyclosome (APC/C), a multi-subunit E3 ubiquitin ligase. Prior to entry into mitosis, the APC/C remains inactive, which allows the accumulation of mitotic regulators. APC/C activation requires binding to either the Cdc20 or Cdh1 adaptor protein, which sequentially bind the APC/C and facilitate targeting of multiple mitotic regulators for proteasomal destruction, including Securin and Cyclin B, to ensure proper chromosome segregation and mitotic exit. Emerging data have indicated that the APC/C, particularly in association with Cdh1, also functions prior to mitotic entry. Specifically, the APC/C-Cdh1 is activated in response to DNA damage in G_2_ phase cells. These observations are in line with in vitro and in vivo genetic studies, in which cells lacking Cdh1 expression display various defects, including impaired DNA repair and aberrant cell cycle checkpoints. In this review, we summarize the current literature on APC/C regulation in response to DNA damage, the functions of APC/C-Cdh1 activation upon DNA damage, and speculate how APC/C-Cdh1 can control cell fate in the context of persistent DNA damage.

## Introduction

Reproduction depends on successful cell division, which is coordinated in the cell cycle. Especially in the context of multicellular organisms, proper control of cell cycle initiation and completion is essential for successful development and homeostasis. Research in the past decades has revealed how cell cycle progression is coordinated by various pathways, which show extensive feedback loops and display multiple levels of cross-talk. Despite this complexity, the core of the cell cycle machinery is constituted by a highly evolutionary conserved molecular ‘engine,’ called the cyclin/cyclin-dependent kinase (CDK) complex [1].

Unicellular eukaryotic organisms, such as the budding yeast *S. cerevisiae,* only have limited genes encoding cyclins and CDKs. In contrast, multicellular organisms, including mammals, express multiple different CDKs, which are often able to bind more than one cyclin, giving rise to a range of distinct cyclin-CDK complexes, each of their activities characterizing discrete phases of the cell cycle.

The activity of CDKs is regulated on multiple transcriptional and post-translational levels. CDK activity is, for instance, extensively controlled through activating (e.g., by the CDK-activating kinase (CAK) [2, 3]) and inhibitory phosphorylation (e.g., by the Wee1 and Myt1 kinases [4–6]). However, probably the most important regulatory layer of oscillating CDK activity relates to the controlled production and down-regulation of their cyclin partners, as CDKs are typically only active when bound to a cyclin. The controlled production and down-regulation of cyclins also holds the key as to how the cell cycle can only progress in a unidirectional fashion, and how S-phase and mitosis are limited to once per cell cycle [7]. The timely destruction of cyclin proteins is accounted for by ubiquitin ligation and ensuing degradation by the 26S proteasome [8]. Ubiquitination of mitotic A- and B-type cyclins is accounted for by the anaphase-promoting complex/cyclosome (APC/C), a multi-subunit E3 ubiquitin ligase. During S and G_2_ phase of the cell cycle, the APC/C remains inactive, which allows the gradual accumulation of mitotic cyclins [9, 10]. Once cells have entered mitosis and have properly aligned their chromosomes, the APC/C is activated and ubiquitinates—among other substrates—mitotic cyclins, and thereby constitutes an important part of the mitotic exit machinery, allowing cells to complete cell division. Through this mechanism, the APC/C forms an integral part of the machinery that ensures periodicity of the cell cycle [8].

Recent evidence has shown that the APC/C also performs additional functions, for instance in response to DNA damage. In this review, we provide a short background on the APC/C and the cellular response to DNA damage. Subsequently, we summarize the current literature on APC/C-Cdh1 activation after DNA damage, and how this affects DNA repair, checkpoint duration, and cell fate.

### Structure and function of the APC/C-Cdh1

The anaphase-promoting complex/cyclosome (APC/C) is an exceptionally large multimeric E3 ubiquitin ligase, belonging to the ring/cullin subfamily of ubiquitin ligases [11, 12]. As it was identified as a protein complex from clam egg extracts that can degrade mitotic cyclins, it was named ‘cyclosome’ [13]. Likewise, a similar 20S complex was biochemically purified from *Xenopus* extracts based on its ability to facilitate cyclin B destruction and to promote anaphase; hence, it was named the ‘anaphase-promoting complex’ (APC) [14]. In parallel, genetic analysis of mutant yeast strains led to the identification of APC components in budding yeast and fission yeast that are required for degradation of Cyclin B and Securin during the metaphase-to-anaphase transition [15–17]. Currently, the term and abbreviation anaphase-promoting complex/cyclosome (APC/C) is used, which also prevents confusion with the frequently mutated tumor suppressor gene *APC*, encoding the Adenomatous Polyposis Coli gene product.

**Fig. 1:**
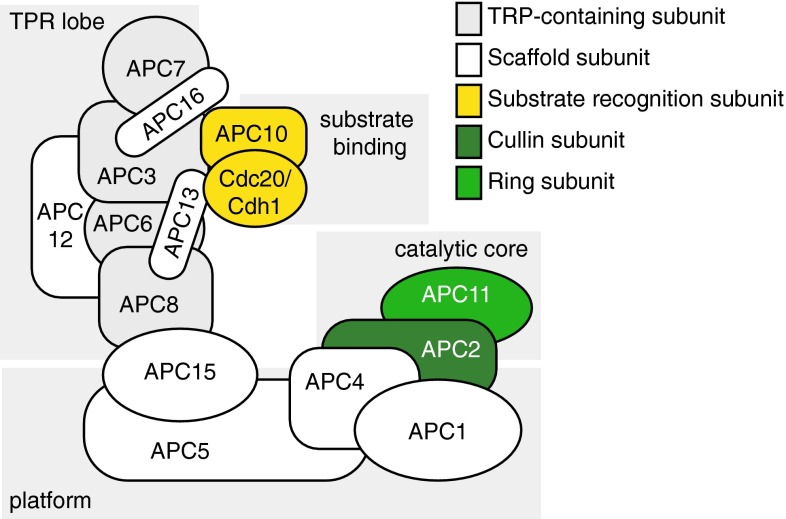
The molecular composition of the APC/C. The subunits of the mammalian APC/C are indicated. APC3, APC6, APC7, and APC8 contain tetracopeptide-repeat (TPR) domains, and together with APC12 for the TPR lobe. APC1, APC4, and APC5 form the ‘platform’ domain of the APC/C. The catalytic core is composed of APC11 (*Ring*) and APC2 (*Cullin*). APC10 together with one the co-activators Cdc20/Cdh1 forms the degron-recognition domain

The APC/C is composed of 14 different subunits in mammalian cells (Table 1), whereas alternative conformations with species-specific subunits have been identified (e.g., the budding yeast-specific subunit APC9, and the fission yeast-specific subunit APC14) [11, 12, 18]. Additionally, the APC/C requires binding to a co-activator (either Cdc20 or Cdh1) for catalytic activity and for substrate recognition. Reconstruction using cryo-electron microscopy recently yielded a complete overview of the 3D assembly of the APC/C [12], and confirmed the triangular ‘shell-like’ shape (Fig. 1), that was proposed based on earlier structural studies. Concerning the stoichiometry of the APC/C, five subunits are present in two copies (APC12 as well as the four tetratricopeptide-repeat (TPR)-containing subunits APC3, APC6, APC7, and APC8), whereas the other subunits are only present in one copy [11, 19]. This conformation leads to an assembly of 19 subunits with a size of 1.22 MDa.

**Table 1:** Human APC/C subunits and co-activators

APC/C subunits	Alternative names	Human gene name	Function
APC1	–	*ANAPC1*	Scaffold, PC repeats
APC2	–	*ANAPC2*	catalytic core-cullin subunit
APC3	Cdc27	*CDC27*	Scaffold with TPR motifs
APC4	–	*ANAPC4*	‘Platform’ subunit
APC5	–	*ANAPC5*	‘Platform’ subunit
APC6	Cdc16	*CDC16*	Scaffold with TPR motifs
APC7	–	*ANAPC7*	Scaffold with TPR motifs
APC8	Cdc23	*CDC23*	Scaffold with TPR motifs
APC10	Doc1	*ANAPC10*	Substrate recognition
APC11	–	*ANAPC11*	Catalytic core-RING H2 domain
APC12	Cdc26	*CDC26*	Stabilizes APC6
APC13	–	*ANAPC13*	Stabilizes APC3, APC6 and APC8
APC15	–	*ANAPC15*	Mediated Cdc20/MCC turnover
APC16	–	*ANAPC16*	TPR subunit

Co-activators	Alternative names	Human gene name	Function
Cdc20	P55-Cdc20	*CDC20*	Substrate recruitment/APC/C activation
Cdh1	Fizzy-related, Hct1 homolog	*FZR1*	Substrate recruitment/APC/C activation

Proteins names, common alternative names, human gene symbols and designated functions of APC/C subunits and co-activators are listed

The ‘back’ of the APC/C complex is also referred to as the ‘TPR’ lobe, as it harbors the four APC/C subunits with conserved tetratricopeptide-repeat motifs (APC3, APC6, APC7, and APC8) as well as the accessory protein APC12. TPR motifs are widely found and consist of 34 amino acids, which promote protein–protein interactions and thereby are often involved in the structural organization of multimeric protein complexes. The TPR-containing subunits within the APC/C are bound and correctly ordered by the APC16 and APC13 subunits (Fig. 1) [12]. The ‘platform’ part of the APC/C is formed by the APC1, APC4, and APC5 subunits [12, 20, 21]. The actual catalytic subunits APC2 (cullin) and APC11 (ring) are located at the periphery of the platform, in close proximity to the degron-recognition module, consisting of APC10 and Cdh1 or Cdc20 (Fig. 1). Binding of the Cdh1/Cdc20 co-activators to the APC/C induces conformational changes in the three-dimensional structure of the APC/C [12], and explains why complex formation between APC/C and Cdh1/Cdc20 elevates catalytic activity [22].

### Activation of the APC/C

Activity of the APC/C is regulated predominantly through binding to its co-activators Cdc20 and Cdh1, as well as through interaction with its inhibitors Emi1 and the MCC [11, 19].

**Fig. 2:**
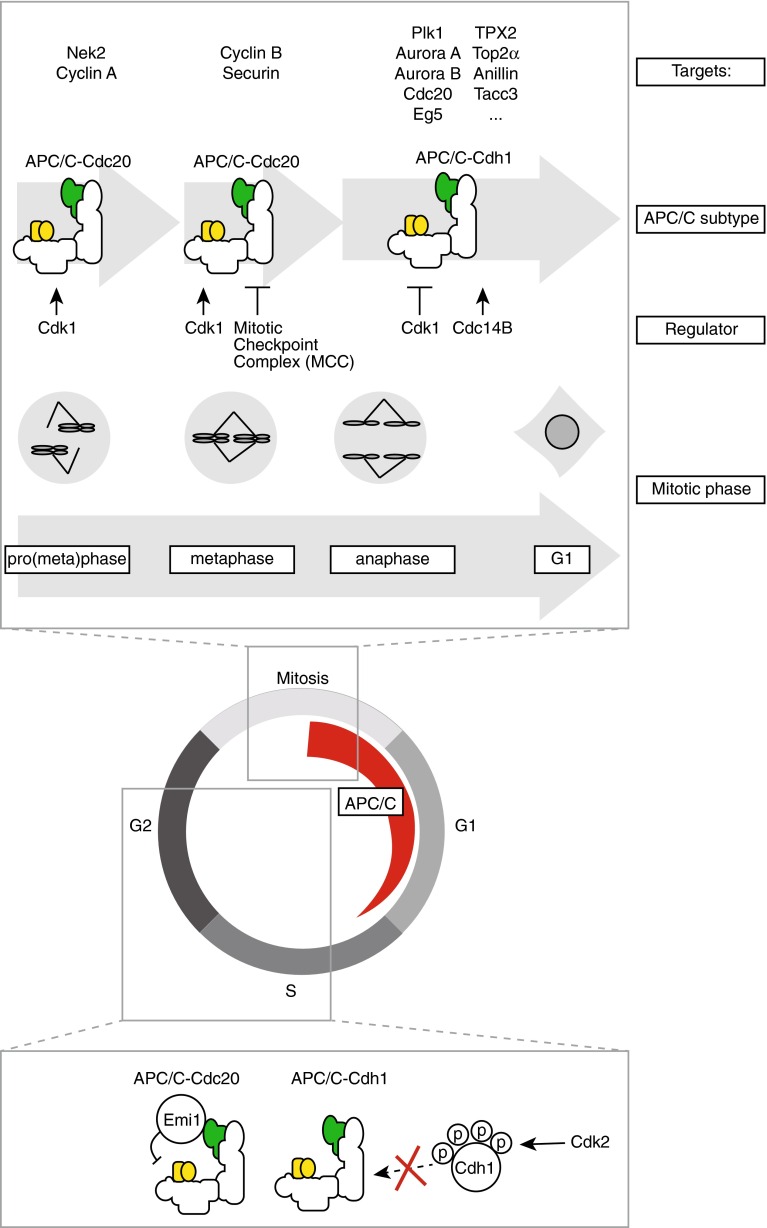
Three waves of APC/C activation during mitosis. The APC/C is activated during mitosis and remains active up until G_1_ phase. Three independent waves of APC/C activity can be distinguished. **1** APC/C-Cdc20 activity during prometaphase, which is unaffected by the spindle checkpoint, **2** APC/C-Cdc20 activity in metaphase, which is under control of the spindle checkpoint, **3** APC/C-Cdh1 activity, which commences during anaphase onset. Identified substrates of each of the APC/C entities are indicated. Interphase activation of the APC/C is precluded by Emi1 and Cdk2-mediated phosphorylation of Cdh1

The co-activators Cdc20 and Cdh1 have a dual role in APC/C-mediated degradation of substrates. First, the WD40 motif in their C-terminal parts is involved in recruitment of substrates to the APC/C. Second, binding of Cdc20 or Cdh1 induces a conformation change in the APC/C to promote ubiquitination. The Cdc20 co-activator associates with the APC/C upon mitotic entry, and remains associated until the metaphase-to-anaphase transition. At that point, Cdc20 is replaced by Cdh1, which remains associated with the APC/C up until G_1_ phase (Fig. 2). Interaction of co-activators with the APC/C is controlled by Cdk1-mediated phosphorylation. Cdc20 phosphorylation by Cdk1 is essentially required for its association with the APC/C and for stimulation of APC/C activity [23, 24]. In contrast, the effect of CDK-mediated phosphorylation on Cdh1, is completely opposite. Cdk1-phosphorylated Cdh1 cannot associate with the APC/C, which effectively limits APC/C-Cdh1 activity until after anaphase onset [23, 24].

Prior to complex formation between Cdh1 and the APC/C, Cdk1-mediated phospho-groups need to be removed, a process that requires phosphatase activity. In budding yeast, Cdh1 dephosphorylation is governed by the Cdc14 phosphatase, under control by the mitotic exit network pathway [25, 26]. In human cells, two Cdc14 isoforms exist, Cdc14A and Cdc14B, and only the Cdc14B isoform appears to be involved in mitotic dephosphorylation of Cdh1 [27] (Fig. 2).

In addition to co-activator binding, which is essential to promote APC/C activation, the APC/C is also subject to inhibition by Emi1 [28]. During interphase, Emi1 serves as a pseudo-APC/C substrate as it binds the APC/C at the D-box receptor and simultaneously interacts with Cdh1 without being degraded. By doing so, Emi1 occupies the substrate binding site of the APC/C to allow accumulation of cyclins [29] (Fig. 2). Upon mitotic entry, phosphorylation of Emi1 by Polo-like kinase-1 (Plk1) creates a phospho-degron on Emi1, which facilitates degradation of Emi1 by the Skp1-Cullin1 F-box (SCF)-β-TrCP complex [28, 30]. Degradation of Emi1 in early mitosis does not actually appear to be required to allow APC/C activation during mitosis, as Emi1 depletion does not interfere with cyclin degradation during mitosis [31]. The main function of Emi1 therefore appears to be safeguarding cyclin accumulation prior to mitosis.

Another inhibitor of the APC/C is the mitotic checkpoint complex (MCC). Until all chromosomes are properly attached to microtubules from the opposing poles of the mitotic spindle, Mad1-Mad2 heterodimers are formed at unattached kinetochores. Once formed, the Mad1-Mad2 dimer induces a change in conformation of a separate Mad2 copy to produce ‘closed-Mad2′ (C-Mad2) [32]. C-Mad2, in conjunction with BubR1 and Bub3, binds and thereby blocks Cdc20. This latter complex is referred to as the MCC, and prevents premature mitotic exit, by inhibiting APC/C-Cdc20-mediated degradation of Cyclin B and Securin until all chromosomes have been properly attached to the mitotic spindle [19, 33–36].

Although, clearly, the APC/C is controlled through binding to activators and inhibitors, post-translational control by phosphorylation is involved in its regulation as well. Multiple subunits of the APC/C are phosphorylated at mitotic onset, including by mitotic kinases Cdk1 and Plk1 [37–39]. Cdk1-mediated phosphorylation events are required for activation of the APC/C-Cdc20, but seem to be dispensable for APC/C-Cdh1 activity [24, 39]. By contrast, phosphorylation of APC/C subunits by Plk1 does not appear to influence the activation of the APC/C-Cdc20 [37, 40, 41]. Nevertheless, phosphorylation of APC/C, as well as its co-activators and inhibitors appears to be a key mechanism in controlling the timing of its activation. And thus, mitotic kinases act in bifurcated ways to ensure rapid activation of the APC/C during mitotic entry.

### Recognition of APC/C substrates

The above-mentioned layers of regulation make that three different phases of APC/C activity can be distinguished, and these phases are best characterized by degradation kinetics of APC/C substrates (Fig. 2). APC/C substrates require ‘degrons,’ specific amino acids sequences that are recognized by APC/C-Cdc20 or APC/C-Cdh1, to be efficiently degraded. Most APC/C substrates contain one or more D-boxes (RXXLXX[I/V]XN) or KEN boxes (KENXXX[N/D]) [42, 43].

A first wave of APC/C activity is observed upon mitotic entry, and depends on the Cdc20 co-activator. During prometaphase, the Cdc20-activated APC/C degrades Cyclin A and the NIMA-related kinase Nek2A [44–47]. Of note, degradation of Cyclin A and Nek2A depends on Cdc20, but is not subject to regulation by the spindle checkpoint. How exactly these early APC/C-Cdc20 targets escape regulation by the MCC is not entirely clear, but the use of alternative degrons appears to be involved.

A second wave of APC/C-Cdc20 activity occurs upon silencing of the spindle checkpoint, and mediates the degradation of Cyclin B and Securin. These two APC/C targets are the only essential targets for cell proliferation [48]. Degradation of Securin is required for sister chromatid separation [49, 50], whereas degradation of Cyclin B is required for exit from mitosis [51–54]. In line with this notion, multiple APC genes as well as the CDC20 gene are essential, whereas Cdh1 is non-essential for survival of individual cells [55–60].

Once the levels of Cyclin B are decreased below a critical level, Cdk1 activity ceases and Cdh1 is allowed to initiate a third wave of APC/C activity. The APC/C-Cdh1 is activated during mitotic exit and targets a range of proteins, including mitotic cyclins regulators of mitotic spindle organization, as well as essential S-phase components [11, 19]. Through suppressing mitotic cyclins, the APC/C-Cdh1 is an important factor in the decision to re-enter a new round of cell division, and ultimately controlling cell fate [61]. In line with this notion, absence of Cdh1 leads to aberrant up-regulation of Cyclin A levels and results in premature initiation of replication. So, although the APC/C-Cdh1 is not essential for cell division of individual cells, this does not mean that the APC/C-Cdh1 does not fulfill an important function, as is also underscored by the lethal phenotype of the Cdh1 knockout mouse [62].

### Cell cycle regulation in situations of DNA damage

As mentioned above, the cell cycle machinery warrants the timely coordination of the various molecular events in which duplication and distribution of chromosomes over daughter cells are managed. In order to ensure that cells are protected against genomic insults, the cell cycle machinery is susceptible to external cues. When DNA is damaged, for instance due to intrinsic factors such as critically short telomeres or replication stress or extrinsic sources including ionizing radiation, cells activate the ‘DNA damage response’ (DDR) [63, 64].

The DDR detects DNA damage, and coordinates a cell cycle arrest with the repair of damaged DNA. Situated upstream in the DDR response, several protein complexes can detect various aberrant DNA structures. For instance, the Mre11/Rad50/Nbs1 (MRN) complex binds the ends of double strand DNA breaks (DSBs) [65], whereas the Rad9-Rad1-Hus1 (9-1-1) complex is loaded onto DNA with structural abnormalities, for instance after UV exposure or stalled replication [66]. Subsequent to detection of DNA lesions, checkpoint kinases are activated. In response to DNA DSBs, the Ataxia Telangiectasia Mutated (ATM) kinase is activated. Conversely, in response to replication stalling, or otherwise generated stretches of single-stranded DNA, the ATM and Rad3-related (ATR) kinase is activated. Both ATM and ATR are members of the PI(3)Kinase-related kinase (PIKK) family, and can phosphorylate an extensive array of substrates, including DNA repair proteins, the RNA translation machinery and metabolic regulators [67]. More importantly, ATM and ATR link the detection of DNA damage to stalling of the cell cycle.

Upon generation of DNA DSBs, ATM phosphorylates and thereby activates the checkpoint kinase Chk2 [68]. In turn, Chk2 phosphorylates members of the Cdc25 phosphatase family which leads to their inhibition [69, 70]. Under normal conditions, the Cdc25 phosphatases counteract the Myt1 and Wee1 kinases, by removing the inhibitory phosphate groups on tyrosine 15 (Y15) and threonine 14 (T14) in Cdk1 and Cdk2 [1, 4, 5, 71, 72]. Consequently, the ATM/Chk2 axis within the DDR prevents Cdc25 activation and keeps Cdk1/2 in a phosphorylated-inactive-state to prevent cell cycle progression. Likewise, ATR phosphorylates the Chk1 checkpoint kinases to achieve similar inactivation of the Cdc25 phosphatases [1–4].

The kinetics of these kinase-driven DDR signaling axes are fast, and mediate a rapid arrest of cell cycle progression within minutes after DNA damage induction [5]. This fast-acting part of the DDR is complemented with a delayed, yet robust, transcriptional pathway. ATM, as well as the ATR, Chk1 and Chk2 kinases, phosphorylate multiple residues within the N-terminus of the p53 transcription factor [6–10]. In parallel, the E3 ubiquitin ligase MDM2, which under physiological conditions ubiquitinates and thereby marks p53 for proteasomal degradation [11], is also phosphorylated by ATM. ATM- or ATR-mediated phosphorylation of MDM2 prevents the association between p53 and MDM2, and leads to stabilization of p53 [12, 13]. In turn, p53 promotes transactivation of its many target genes, including the cyclin-dependent kinase inhibitor (CKI) p21^cip/waf^ [14, 15] and the cell cycle checkpoint protein Gadd45 [16, 17]. Although this transcriptional DNA damage response requires hours to be installed, its downstream effects can last for days. The combination of a kinase-driven fast-acting DDR axis with a delayed transcriptional DNA damage response thus constitutes a robust cellular reaction to maintain genomic integrity. Temporarily arresting cell cycle progression can provide time for repair of DNA lesions, after which cells can silence checkpoints to restart the cell cycle [18, 19].

When the levels of DNA damage are beyond repair, however, affected cells need to be permanently arrested (senescence) or eliminated through programmed cell death (apoptosis). Failure to do so is associated with increased tumorigenesis [20]. How exactly cells make the decision to enter a permanent cell cycle arrest after DNA damage is unclear. Recent data have shown that entrance into senescence is dependent on the level of DNA damage, is p53-dependent and can happen both in G_1_ and G_2_ cells [21, 22].

### Recently emerging roles of the APC/C-Cdh1 in the response to DNA damage

In line with a role of the APC/C-Cdh1 in degradation of mitotic regulators, inactivation of the *Cdh1* locus in chicken DT40 cells resulted in accumulation of mitotic cyclins in G_1_ cells [23]. Unexpectedly, *Cdh1* knock-out cells failed to maintain a DNA damage-induced G_2_ cell cycle checkpoint arrest [23]. These data suggested for the first time that the APC/C-Cdh1 also has a function in G_2_ phase of the cell cycle. This role, however, seems to be restricted to situations in which there is DNA damage. Indeed, upon irradiation, Cdh1 was shown to associate with the APC/C, using co-immunoprecipitation assays in cell line models from several species [23]. Moreover, purified APC/C from irradiated G_2_ cells was activated when assessed using in vitro ubiquitination assays toward Cdc20 [23].

Under normal conditions, the APC/C-Cdh1 is unable to ubiquitinate substrates in G_2_ phase and early mitosis. This is achieved through multiple mechanisms. First, CDK-mediated phosphorylation of Cdh1 occurs on different residues prior to the metaphase-to-anaphase transition, and these phosphorylation events prevent association of Cdh1 with the APC/C [24, 25]. Importantly, a Cdh1 mutant in which CDK phosphorylation sites were removed activated the APC/C already in S-phase [25, 26]. Likewise, depletion of Cyclin A also prematurely activated the APC/C-Cdh1, suggesting that a Cyclin A/CDK complex is required to keep APC/C-Cdh1 inactive during interphase [26]. Second, as also explained previously, the APC/C is kept inactive during S and G_2_ phase by Emi1 [27, 28]. Emi1 is expressed from late G_1_ onwards, and sterically inhibits the APC/C [27, 29]. Only during mitotic entry, Emi1 is degraded by the SCF-β-TrCP [30–32]. The impact of Emi1-mediated APC/C inhibition becomes apparent after Emi1 depletion: Cyclins A and B do not accumulate, and cells do not initiate S-phase, nor enter mitosis [28, 29].

### Activation of the APC/C-Cdh1 in response to DNA damage; How is it accomplished?

Although genetic and biochemical evidence was provided which indicated that the APC/C-Ch1 can be activated in response to DNA damage, it is not entirely clear how this is accomplished mechanistically. Most evidence so far points at regulation of the phosphorylation status of Cdh1. The activity of cyclin/CDK complexes is down-regulated after activation of the DDR, through rapid inactivation of the Cdc25 phosphatases that under normal circumstances activate CDKs. However, the level of CDK inactivation that the kinase-driven DDR axis accomplishes does not seem to be sufficient for APC/C-Cdh1 activation [33]. Rather, the p53/p21 transcriptional DDR axis is required for APC/C-Cdh1 activation, since deletion of *TP53* or *CDKN1A* (encoding p21) abrogated APC/C-Cdh1 activation after DNA damage [33]. Whether the function of p53 in this context is solely to lower CDK activity is unclear, as it also leads to down-regulation of Emi1 and could through this effect also promote activation of the APC/C-Cdh1 [33].

Besides regulation of CDK kinase activity, the phosphatase that removes CDK-mediated phosphorylation groups appears differentially regulated upon DNA damage as well. In budding yeast, the Cdc14 phosphatase is involved in reversing CDK phosphorylation events during anaphase [34], controlled by the Mitotic Exit Network (MEN) pathway [35]. As part of this mechanisms, Cdc14 is released from the nucleolus to promote Cdh1 dephosphorylation and ensuing APC/C activation [34, 36]. Human cells contain two Cdc14 orthologues: Cdc14A and Cdc14B. Of these two, Cdc14B is localized to nucleoli, from which it is released during mitosis [37]. In contrast to yeast, however, Cdc14B in human cells is not essentially required for mitotic exit [37]. Interestingly, in G_2_ cells Cdc14B is released from the nucleolus in response to DNA damage [38, 39].

Combined, it seems that three events contribute to APC/C-Cdh1 activation in G_2_ cells: (1) down-regulation of CDK activity in response to DDR activation, (2) p53-dependent inactivation of APC/C inhibitor Emi1, and (3) the translocation of Cdc14B phosphatase from the nucleolus to the nucleoplasm. Many questions about the exact molecular wiring of these pathways, however, remain unsolved [39]. For instance: is Emi1 degradation required for APC/C-Cdh1 activation? Another question relates to how Cdc14B is released from the nucleolus in response to DNA damage. More interestingly perhaps, it remains unclear whether these various events are connected.

Although unexplored, a more direct way of APC/C regulation concerns direct modification of APC/C subunits in response to DNA damage, through direct phosphorylation by ATM/ATR. In a large proteomics study to identify ATM and ATR substrates, APC12 was found to be phosphorylated on Ser-78 in response to ionizing radiation [40]. Although it is unclear what the functional impact of this phosphorylation event is, this residue appears conserved in mammals, but not in all vertebrates, such as chicken and frog (data not shown). Although Cdc26/APC12 does not appear to be directly involved in catalytic activity of the APC/C, modification of the APC/C in response to DNA damage might alter the conformation of the APC/C, or possibly have an impact on its activity or substrate specificity.

### Regulation of the G_2_/M checkpoint by the APC/C-Cdh1

Activation of the APC/C-Cdh1 appears to contribute to the maintenance of a DNA damage-induced G_2_/M cell cycle checkpoint. Cdh1 inactivation in DT40 chicken cells leads to premature checkpoint termination [23]. Although the APC/C-Cdh1 has many reported targets, the defects in G_2_/M checkpoint maintenance were attributed to defective down-regulation of Plk1. Out of 15 assessed APC/C-Cdh1 targets, only Plk1 showed APC/C-Cdh1-dependent degradation in response to DNA damage. Specifically, expression of a Plk1 mutant that cannot be targeted by the APC/C resulted in enhanced mitotic entry in the presence of DNA damage [38].

The surprising finding that not all APC/C-Cdh1 targets are down-regulated after DNA damage was at least in part explained by the action of the deubiquitinating (DUB) enzyme USP28. The APC/C-Cdh1 target Claspin, for instance, is protected from APC/C-mediated down-regulation by USP28 [38], and also 53BP1 and Chk2 were reported to be stabilized by USP28 upon DNA damage [41]. An additional explanation for selective substrate engagement of the APC/C-Cdh1 would be the presence of two distinct pools of APC/C [42]. One pool presumably is inactive in G_2_, regardless of the presence of DNA damage, by virtue of inhibition by Emi1. A second pool would be inactive in G_2_ cells due to CDK-mediated phosphorylation. Only this second pool presumably becomes active upon DNA damage, due to Cdc14B-mediated dephosphorylation of Cdh1 and/or down-regulation of CDK activity [42]. This model is supported by the observation that USP28 depletion does not lead to general degradation of APC/C-Cdh1 targets, and the observation that Emi1 association with the APC/C does not appear to be controlled by DNA damage [38].

The APC/C-Cdh1 also targets other proteins that are crucial for the G_2_/M transition, which could explain the effects of Cdh1 depletion on checkpoint maintenance. The MEF2C and FoxM1 transcription factors, for instance, are Cdh1 targets and promote the expression of multiple cell cycle genes including 14-3-3γ, Gadd45b, and p21 for MEF2C; and Cyclin B1 and Plk1 for FoxM1 [43–45]. Whether MEF2C and FoxM1 are degraded in a Cdh1-dependent fashion in response to DNA damage, however, is not known. Another APC/C-Cdh1 target is the Wip1 phosphatase [46]. Wip1 can reverse ATM/ATR-mediated phosphorylation groups, for instance on H2AX and p53, and is involved in the restart of the cell cycle when DNA has been repaired [47–50]. Proteolytic down-regulation of Wip1 could aid in maintaining a cell cycle arrest in situations of DNA damage, although this hypothesis has yet to be confirmed experimentally.

### Regulation of DNA repair by the APC/C-Cdh1

In addition to its effects on G_2_ checkpoint behavior, the APC/C-Cdh1 has also been implicated in modulating DNA repair.

Concerning the repair of DNA double strand breaks (DSBs), cells can choose between two fundamentally different repair pathways. Repair through error prone non-homologous end-joining (NHEJ) can occur throughout the cell cycle, and ligates DNA ends in a sequence-independent fashion [51]. Alternatively, cells can repair DSBs using homologous recombination (HR). Since this process uses sister chromatids as a template for error-free repair, HR is restricted to S/G_2_ phase of the cell cycle [52]. A key step in HR is the formation of single-stranded (ss)DNA through 5’-to-3’ end resection. This process is facilitated by the MRN complex, in conjunction with CtIP [53]. As DNA ends with long ssDNA tails are not substrates for end-joining repair, the process of DNA end resection is the key decisive point where repair is committed to HR repair.

In fission yeast, the Rad54 homolog rhp54 was shown to be a target of the APC/C-Cdh1 [54]. Rad54 is a DNA-dependent ATPase and is critically required for HR. Expression of a non-degradable Rhp54 mutant leads to aberrant HR and to increased sensitivity to a range of DNA damaging agents, including bleomycin and UV [54]. However, no APC/C-dependent degradation of the *S. cerevisiae* Rad54 nor human Rad54A or Rad54B was observed, suggesting the observed phenotype is species-specific [54]. Upstream in the HR pathways, the Receptor-Associated Protein 80 (Rap80) forms a complex with Brca1, and facilitates the recruitment of Brca1 to sites of DNA damage [55]. During mitotic exit, Rap80 is degraded in an APC/C-dependent fashion, which is thought to prevent illegitimate recombination during G_1_ phase of the cell cycle [56].

In a proteomic screen, the homologous recombination DNA repair protein CtIP was identified as a APC/C-Cdh1 target [57]. Specifically, CtIP was down-regulated in an APC/C-Cdh1-dependent manner, both during mitotic exit as well as in response to DNA damage. Notably, expression of a non-degradable CtIP mutant resulted in extended retention of CtIP at sites of DNA damage, caused elevated levels of DNA end resection, and ultimately interfered with normal DNA repair through recombination [57]. From these data, it appears that APC/C activation at late time points after DNA damage is required for proper DNA repair. This observation is in line with data from Cdc14B knockout cells. Loss of Cdc14B, which should lead to an inability to activate the APC/C-Cdh1 in response to DNA damage, resulted in defective DNA repair [58].

The APC/C-Cdh1 has also been implicated in controlling replication. Prior to S-phase initiation, the de-ubiquitinating enzyme USP1 is down-regulated by the APC/C-Cdh1, which allows PCNA to be mono-ubiquitinated in response to UV [59]. Thus, the APC/C-Cdh1 is required to equip cells with the ability to deal with potential replication-blocking lesions in the ensuing S-phase. During replication stress, exposed ssDNA activates the ATR kinase, which phosphorylates the Rad17 protein [60, 61]. Subsequently, Rad17 is thought to be involved in the loading of the Rad9-Rad1-Hus1 (9-1-1) checkpoint-sliding clamp onto DNA, as well as the activation of the Claspin/Chk1 complex to facilitate cell cycle checkpoint activation and repair [62, 63]. Rad17 was shown to be degraded in response to UV exposure, in an APC/C-Cdh1-dependent fashion [64]. Rather than controlling DNA repair, damage-induced degradation of Rad17 appears to be required for cell cycle re-entry [64].

Other APC/C-Cdh1-mediated effects during the response to replication stress include the stabilization of the Cdc7, as well as its co-factor: activator of S-phase kinase (ASK, also referred to as Dfb4). In response to a hydroxyurea-induced replication arrest, Cdc7 and ASK are stabilized. Cdc7/ASK activity, in turn, stimulates DNA lesion bypass, also known as translesion synthesis (TLS) [65]. Mechanistically, Cdh1 was shown to promote its own degradation, in a manner that was stimulated by Chk1. How exactly Chk1 promotes the inactivation of Cdh1 is not clear [65]. Also the APC/C-Cdh1 target PCNA-associated factor-15 (p15-PAF) is involved in TLS [66]. In response to replication stalling, p15-PAF is removed from chromatin by proteasomal degradation. Notably, whereas the APC/C-Cdh1 is responsible for p15-PAF degradation during G_1_ phase of the cell cycle [67, 68], a different ubiquitin ligase is responsible for p15-PAF degradation upon replication stress, as this process is independent of its APC/C destruction boxes [66]. Additional genes that function in S-phase checkpoint pathways, and were shown to be targeted by the APC/C, include Tos4 and Pdr3 in budding yeast, although the implications of APC/C-mediated regulation of these proteins remains to be established [69].

### APC/C and Cdh1 in cancer

Proper cell cycle control is key in preventing oncogenic transformation. It has been long recognized that Cdh1 is instrumental in establishing the G_1_ phenotype after a round of cell division. Cdh1 is required to down-regulate components of the replication and mitotic spindle machinery, and APC/C-Cdh1 activity in G_1_ phase prevents the accumulation of essential genes for another round of DNA replication. In line with this notion, inactivation of mouse *Fzr1* gene (encoding Cdh1) prevented the terminal differentiation of neuronal progenitors and resulted in increased proliferation of neuronal stem cells, and conversely, defective neurogenesis [70, 71]. As a consequence of unscheduled S-phase entry, Cdh1 loss leads to replication stress and ensuing genomic instability [72], a commonly recognized driver of oncogenesis. The latter observations underscore the potential tumor-suppressive function of Cdh1. In good agreement with this model, Cdh1 inactivation leads to the development of several cancers in mice [71], and low Cdh1 expression was observed in multiple cancer cell lines [73]. Also, the *PTEN* tumor suppressor gene was shown to stimulate association of Cdh1 with the APC/C [74], and hence, the frequently observed loss of *PTEN* may indirectly result in impaired APC/C-Cdh1 function in cancers. Likewise, the tumor-suppressor sirtuin gene *SIRT2* was shown to promote APC/C-Cdh1 function through acetylation of Cdh1, and again may explain tumor-suppresive effects of indirect APC/C-Cdh1 inactivation [75].

Whether the tumor-suppressive function of Cdh1 also involves its role in controlling cell fate after DNA damage is unclear. The observation that APC/C-Cdh1 degrades the histone methyltransferases G9a and GLP in response to DNA damage may point at such a role [76]. Specifically, G9a and GLP are degraded by the APC/C-Cdh1 in a Cdc14B- and p21-dependent fashion. Both G9a and GLP are responsible for positioning histoneH3-Lysine-9 mono- and di-methyl marks, epigenetic marks of gene silencing. Consequently, DNA damage leads to a global decrease of H3K9 methylation, along with elevated expression of IL6 and IL8, two interleukins that contribute to the senescence phenotype [76]. Importantly, this function of the APC/C was uncovered in response to oncogene-induced replication stress, indicating that Cdh1 prevents outgrowth of cells with oncogene-induced DNA damage. Therapeutically, the increased levels of replication stress in cancer cells with inactivated Cdh1 may create sensitivity to targeted agents that target the replication checkpoint, such as inhibitors of Wee1, ATR or Chk1. In contrast, loss of Cdh1 was recently shown to bypass the dependency of cancer cells on Cdk4/6 [77], indicating that Cdh1 loss may constitute an exclusion criterion for treatment with the recently developed Cdk4/6 inhibitors, or could be a mechanism of acquired resistance to such drugs.

Although some cancer cell lines were reported to have loss of Cdh1 expression, analysis of TCGA data shows that APC/C components and Cdh1 are not commonly inactivated in human cancers. This may point at an essential role of the APC/C and/or APC/C cofactors for the growth of some cancers. Indeed, using an siRNA-based screening method, loss of APC/C activity was shown to be synthetic lethal with loss of chromosome cohesion. As a consequence, therapeutic targeting of the APC/C may be beneficial in cohesion-defective cancers [78]. In this context, it is important that small molecule inhibitors of the APC/C have been developed (proTAME and APCin), which in combination block total APC/C activity [79].

### Concluding remarks

**Fig. 3:**
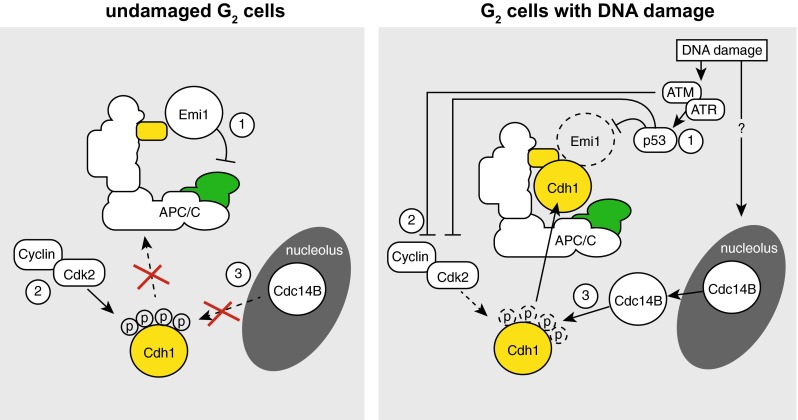
Roles of the APC/C-Cdh1 in response to DNA damage. During an unperturbed interphase, the APC/C is not active due to **1** binding of Emi1, **2** phosphorylation of Cdh1 by Cdk2, and **3** the inability of Cdc14B to dephosphorylate Cdh1. In response to DNA damage in G_2_ cells, **1** the DDR kinases ATM and ATR mediate activation of p53, which leads to Emi1 down-regulation. **2** ATM/ATR and p53 inactivate Cdk2 activity, and **3** Cdc14B is released from the nucleolus through unknown mechanisms. Combined, these mechanisms lead to activation of the APC/C-Cdh1

**Fig. 4:**
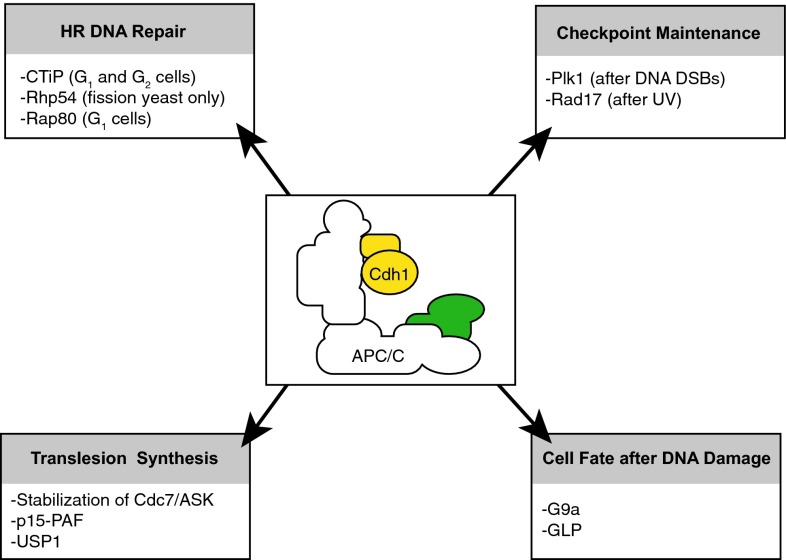
Functions of the APC/C-Cdh1 in situations of DNA damage. Different functions of the APC/C-Cdh1 and its targets are illustrated

Increasingly, the APC/C-Cdh1 is recognized to be involved in the cellular response to DNA damage. The contours of the mechanisms that underlie APC/C-Cdh1 activation have become apparent, with Cdc14B and the p53/p21 axis being clearly involved (Fig. 3). Of note, many studies in this area employ high levels of DNA damage, which exceed physiological levels of DNA lesions, and may only reflect situations of cancer treatment with genotoxic agents. Nevertheless, recent studies using oncogene-induced DNA damage and persistent telomere damage confirmed the activation of the APC/C-Cdh1 [76, 80], and established similar genetic requirement, i.e., the presence of Cdc14 and p53/p21 [76]. Finally, the observation that Cdh1 inactivation leads to high levels of genomic instability and accumulation, underscores the relevance of Cdh1 in maintaining genomic integrity even in unchallenged conditions [71, 81, 82] (Fig. 4).

Concluding, the APC/C-Cdh1 constitutes one of the effector pathways of the response to DNA damage. Further research is warranted to better understand the mechanisms that underpin APC/C-Cdh1 activation after DNA damage, and which consequences it has. Uncovering these regulatory mechanisms and phenotypes may provide further insight into how cells are wired to cope with genomic stress, and how these mechanisms may be altered during tumorigenesis and can potentially be exploited during cancer treatment.
